# Supplementary material for: Modeling glioblastoma heterogeneity as a dynamic network of cell states
Source: Mol Syst Biol. 2021 Sep 16;17(9):e10105. doi: 10.15252/msb.202010105 (PMC8444284; doi:10.15252/msb.202010105)
Supplement: Supplementary file 5 — Source Data for Figure 3 [file MSB-17-e10105-s001.zip › Figure3A_sourcedata/GSEA_3065/nosigdb_state5.GseaPreranked.1622718435914/RICHARDS_NOWAKOWSKI_SCIENCE_2017_ASTROCYTE_UPREG.html]

Details for gene set RICHARDS\_NOWAKOWSKI\_SCIENCE\_2017\_ASTROCYTE\_UPREG[GSEA]

|  || Dataset | state5 |
| Phenotype | NoPhenotypeAvailable |
| Upregulated in class | na\_pos |
| GeneSet | RICHARDS\_NOWAKOWSKI\_SCIENCE\_2017\_ASTROCYTE\_UPREG |
| Enrichment Score (ES) | 0.41774604 |
| Normalized Enrichment Score (NES) | 1.9813026 |
| Nominal p-value | 0.0 |
| FDR q-value | 0.0021545896 |
| FWER p-Value | 0.011 |
Table: GSEA Results Summary

  

Fig 1: Enrichment plot: RICHARDS\_NOWAKOWSKI\_SCIENCE\_2017\_ASTROCYTE\_UPREG      
 Profile of the Running ES Score & Positions of GeneSet Members on the Rank Ordered List

  

| PROBE | GENE SYMBOL | GENE\_TITLE | RANK IN GENE LIST | RANK METRIC SCORE | RUNNING ES | CORE ENRICHMENT || 1 | MALAT1 |  |  | 0 | 1.196 | 0.0108 | Yes |
| 2 | NOVA1 |  |  | 10 | 0.870 | 0.0175 | Yes |
| 3 | PTPRZ1 |  |  | 12 | 0.862 | 0.0251 | Yes |
| 4 | WSB1 |  |  | 13 | 0.861 | 0.0329 | Yes |
| 5 | CD9 |  |  | 14 | 0.857 | 0.0406 | Yes |
| 6 | BCAN |  |  | 15 | 0.850 | 0.0482 | Yes |
| 7 | NEAT1 |  |  | 17 | 0.830 | 0.0556 | Yes |
| 8 | PTN |  |  | 19 | 0.824 | 0.0629 | Yes |
| 9 | CD63 |  |  | 24 | 0.769 | 0.0693 | Yes |
| 10 | HSP90B1 |  |  | 33 | 0.729 | 0.0749 | Yes |
| 11 | SCRG1 |  |  | 36 | 0.715 | 0.0811 | Yes |
| 12 | GPM6B |  |  | 41 | 0.702 | 0.0870 | Yes |
| 13 | TNC |  |  | 45 | 0.680 | 0.0927 | Yes |
| 14 | CANX |  |  | 53 | 0.634 | 0.0976 | Yes |
| 15 | GPM6A |  |  | 60 | 0.609 | 0.1024 | Yes |
| 16 | B2M |  |  | 61 | 0.601 | 0.1078 | Yes |
| 17 | TMBIM6 |  |  | 68 | 0.584 | 0.1123 | Yes |
| 18 | DDR1 |  |  | 81 | 0.551 | 0.1158 | Yes |
| 19 | SLC1A3 |  |  | 82 | 0.550 | 0.1208 | Yes |
| 20 | PON2 |  |  | 84 | 0.546 | 0.1256 | Yes |
| 21 | FGFBP3 |  |  | 85 | 0.545 | 0.1305 | Yes |
| 22 | MSMO1 |  |  | 92 | 0.540 | 0.1346 | Yes |
| 23 | LAPTM4B |  |  | 97 | 0.532 | 0.1389 | Yes |
| 24 | TTC14 |  |  | 100 | 0.526 | 0.1434 | Yes |
| 25 | LRRC4C |  |  | 101 | 0.525 | 0.1482 | Yes |
| 26 | CADM4 |  |  | 102 | 0.525 | 0.1529 | Yes |
| 27 | PRCP |  |  | 103 | 0.523 | 0.1576 | Yes |
| 28 | PLEKHH2 |  |  | 106 | 0.520 | 0.1620 | Yes |
| 29 | DSEL |  |  | 107 | 0.518 | 0.1667 | Yes |
| 30 | TIMP1 |  |  | 109 | 0.518 | 0.1712 | Yes |
| 31 | SEMA5A |  |  | 111 | 0.516 | 0.1757 | Yes |
| 32 | ITM2B |  |  | 117 | 0.509 | 0.1797 | Yes |
| 33 | ITGAV |  |  | 118 | 0.508 | 0.1843 | Yes |
| 34 | ITGB8 |  |  | 126 | 0.503 | 0.1880 | Yes |
| 35 | TMED10 |  |  | 131 | 0.498 | 0.1920 | Yes |
| 36 | LSAMP |  |  | 132 | 0.497 | 0.1965 | Yes |
| 37 | IGFBP2 |  |  | 137 | 0.491 | 0.2004 | Yes |
| 38 | TTYH1 |  |  | 141 | 0.489 | 0.2045 | Yes |
| 39 | PNISR |  |  | 150 | 0.482 | 0.2078 | Yes |
| 40 | CLSTN1 |  |  | 154 | 0.478 | 0.2118 | Yes |
| 41 | FAT1 |  |  | 155 | 0.478 | 0.2161 | Yes |
| 42 | PPIB |  |  | 156 | 0.477 | 0.2204 | Yes |
| 43 | CD81 |  |  | 159 | 0.475 | 0.2244 | Yes |
| 44 | DPP6 |  |  | 161 | 0.475 | 0.2286 | Yes |
| 45 | CLU |  |  | 162 | 0.474 | 0.2328 | Yes |
| 46 | ABHD2 |  |  | 163 | 0.473 | 0.2371 | Yes |
| 47 | MMP16 |  |  | 165 | 0.470 | 0.2412 | Yes |
| 48 | SCD5 |  |  | 173 | 0.460 | 0.2445 | Yes |
| 49 | PDGFRA |  |  | 175 | 0.460 | 0.2485 | Yes |
| 50 | SLC16A1 |  |  | 186 | 0.449 | 0.2514 | Yes |
| 51 | MAGI1 |  |  | 196 | 0.442 | 0.2543 | Yes |
| 52 | RCN1 |  |  | 217 | 0.427 | 0.2558 | Yes |
| 53 | MAML2 |  |  | 220 | 0.425 | 0.2594 | Yes |
| 54 | ALCAM |  |  | 225 | 0.422 | 0.2627 | Yes |
| 55 | PSAP |  |  | 227 | 0.420 | 0.2663 | Yes |
| 56 | SYPL1 |  |  | 231 | 0.418 | 0.2698 | Yes |
| 57 | MFGE8 |  |  | 233 | 0.418 | 0.2734 | Yes |
| 58 | SERPINE2 |  |  | 238 | 0.414 | 0.2766 | Yes |
| 59 | PTPRG |  |  | 245 | 0.411 | 0.2796 | Yes |
| 60 | LRP1 |  |  | 259 | 0.404 | 0.2817 | Yes |
| 61 | SYT11 |  |  | 265 | 0.400 | 0.2847 | Yes |
| 62 | SCD |  |  | 284 | 0.391 | 0.2861 | Yes |
| 63 | PCDH9 |  |  | 285 | 0.390 | 0.2896 | Yes |
| 64 | HSD17B12 |  |  | 311 | 0.377 | 0.2901 | Yes |
| 65 | DST |  |  | 313 | 0.376 | 0.2934 | Yes |
| 66 | NRBP2 |  |  | 322 | 0.372 | 0.2958 | Yes |
| 67 | TMEM132B |  |  | 335 | 0.365 | 0.2976 | Yes |
| 68 | FKBP10 |  |  | 337 | 0.365 | 0.3008 | Yes |
| 69 | SFXN5 |  |  | 342 | 0.364 | 0.3036 | Yes |
| 70 | NOTCH1 |  |  | 348 | 0.361 | 0.3063 | Yes |
| 71 | LGALS3BP |  |  | 349 | 0.361 | 0.3095 | Yes |
| 72 | RNF180 |  |  | 357 | 0.359 | 0.3119 | Yes |
| 73 | FJX1 |  |  | 371 | 0.352 | 0.3135 | Yes |
| 74 | DAG1 |  |  | 373 | 0.351 | 0.3166 | Yes |
| 75 | SPPL2A |  |  | 396 | 0.343 | 0.3171 | Yes |
| 76 | SEZ6L |  |  | 422 | 0.334 | 0.3171 | Yes |
| 77 | BCHE |  |  | 425 | 0.333 | 0.3199 | Yes |
| 78 | PCDHGC3 |  |  | 428 | 0.331 | 0.3226 | Yes |
| 79 | TMEM106B |  |  | 435 | 0.330 | 0.3249 | Yes |
| 80 | UFL1 |  |  | 436 | 0.330 | 0.3279 | Yes |
| 81 | GPC6 |  |  | 443 | 0.326 | 0.3301 | Yes |
| 82 | MTRNR2L8 |  |  | 447 | 0.326 | 0.3327 | Yes |
| 83 | TIMP2 |  |  | 451 | 0.325 | 0.3352 | Yes |
| 84 | SERPINH1 |  |  | 460 | 0.322 | 0.3372 | Yes |
| 85 | AASS |  |  | 494 | 0.313 | 0.3361 | Yes |
| 86 | RFX4 |  |  | 509 | 0.308 | 0.3373 | Yes |
| 87 | MLEC |  |  | 518 | 0.306 | 0.3391 | Yes |
| 88 | EDNRB |  |  | 519 | 0.306 | 0.3418 | Yes |
| 89 | COL6A1 |  |  | 536 | 0.301 | 0.3426 | Yes |
| 90 | FAM3C |  |  | 538 | 0.301 | 0.3452 | Yes |
| 91 | ITM2C |  |  | 551 | 0.297 | 0.3465 | Yes |
| 92 | ITGA6 |  |  | 552 | 0.297 | 0.3492 | Yes |
| 93 | FGFR3 |  |  | 567 | 0.293 | 0.3501 | Yes |
| 94 | SSR3 |  |  | 575 | 0.291 | 0.3519 | Yes |
| 95 | LAMB2 |  |  | 578 | 0.290 | 0.3543 | Yes |
| 96 | HIBCH |  |  | 585 | 0.289 | 0.3562 | Yes |
| 97 | NTRK2 |  |  | 587 | 0.288 | 0.3587 | Yes |
| 98 | MLC1 |  |  | 591 | 0.288 | 0.3609 | Yes |
| 99 | SLC4A4 |  |  | 598 | 0.286 | 0.3628 | Yes |
| 100 | PLOD2 |  |  | 613 | 0.282 | 0.3637 | Yes |
| 101 | IFNAR1 |  |  | 614 | 0.282 | 0.3662 | Yes |
| 102 | CPE |  |  | 624 | 0.280 | 0.3677 | Yes |
| 103 | IL6ST |  |  | 627 | 0.279 | 0.3699 | Yes |
| 104 | PMP22 |  |  | 663 | 0.272 | 0.3683 | Yes |
| 105 | DNER |  |  | 671 | 0.270 | 0.3699 | Yes |
| 106 | ZMYM2 |  |  | 673 | 0.270 | 0.3722 | Yes |
| 107 | JAM2 |  |  | 675 | 0.270 | 0.3745 | Yes |
| 108 | LRP1B |  |  | 676 | 0.269 | 0.3769 | Yes |
| 109 | TNK2 |  |  | 681 | 0.268 | 0.3788 | Yes |
| 110 | ATP9B |  |  | 687 | 0.267 | 0.3807 | Yes |
| 111 | SPECC1 |  |  | 689 | 0.266 | 0.3829 | Yes |
| 112 | HTRA1 |  |  | 690 | 0.266 | 0.3853 | Yes |
| 113 | ZBTB20 |  |  | 696 | 0.263 | 0.3871 | Yes |
| 114 | GOLIM4 |  |  | 699 | 0.262 | 0.3892 | Yes |
| 115 | DAD1 |  |  | 721 | 0.257 | 0.3891 | Yes |
| 116 | SDC3 |  |  | 728 | 0.255 | 0.3906 | Yes |
| 117 | ADAMTS6 |  |  | 733 | 0.254 | 0.3925 | Yes |
| 118 | CADM2 |  |  | 739 | 0.253 | 0.3941 | Yes |
| 119 | RNF13 |  |  | 745 | 0.251 | 0.3958 | Yes |
| 120 | LRIG1 |  |  | 754 | 0.250 | 0.3971 | Yes |
| 121 | NOTCH2 |  |  | 756 | 0.249 | 0.3992 | Yes |
| 122 | FADS2 |  |  | 780 | 0.245 | 0.3987 | Yes |
| 123 | MCFD2 |  |  | 786 | 0.244 | 0.4003 | Yes |
| 124 | IDH1 |  |  | 793 | 0.242 | 0.4018 | Yes |
| 125 | STAT3 |  |  | 797 | 0.242 | 0.4036 | Yes |
| 126 | PNPT1 |  |  | 803 | 0.240 | 0.4052 | Yes |
| 127 | OLIG1 |  |  | 809 | 0.239 | 0.4068 | Yes |
| 128 | TSPAN3 |  |  | 822 | 0.237 | 0.4075 | Yes |
| 129 | GPRC5B |  |  | 838 | 0.234 | 0.4078 | Yes |
| 130 | PRNP |  |  | 851 | 0.232 | 0.4085 | Yes |
| 131 | SPATA6 |  |  | 852 | 0.232 | 0.4106 | Yes |
| 132 | PTPRA |  |  | 855 | 0.232 | 0.4124 | Yes |
| 133 | NTRK3 |  |  | 908 | 0.224 | 0.4083 | Yes |
| 134 | COMT |  |  | 938 | 0.220 | 0.4069 | Yes |
| 135 | IFI6 |  |  | 961 | 0.216 | 0.4063 | Yes |
| 136 | LDLRAD3 |  |  | 964 | 0.216 | 0.4080 | Yes |
| 137 | ILDR2 |  |  | 965 | 0.215 | 0.4099 | Yes |
| 138 | PDE4B |  |  | 968 | 0.215 | 0.4116 | Yes |
| 139 | ZDHHC21 |  |  | 984 | 0.212 | 0.4118 | Yes |
| 140 | TRPS1 |  |  | 1004 | 0.210 | 0.4114 | Yes |
| 141 | ST3GAL5 |  |  | 1032 | 0.206 | 0.4101 | Yes |
| 142 | TAPBP |  |  | 1047 | 0.204 | 0.4103 | Yes |
| 143 | MEGF10 |  |  | 1049 | 0.204 | 0.4120 | Yes |
| 144 | C1orf61 |  |  | 1057 | 0.204 | 0.4130 | Yes |
| 145 | SEMA6A |  |  | 1075 | 0.202 | 0.4128 | Yes |
| 146 | SLC35F1 |  |  | 1092 | 0.199 | 0.4127 | Yes |
| 147 | SC5D |  |  | 1094 | 0.199 | 0.4144 | Yes |
| 148 | LRRN1 |  |  | 1103 | 0.198 | 0.4152 | Yes |
| 149 | NLGN4X |  |  | 1136 | 0.194 | 0.4132 | Yes |
| 150 | STK3 |  |  | 1150 | 0.191 | 0.4134 | Yes |
| 151 | ZNF236 |  |  | 1167 | 0.189 | 0.4132 | Yes |
| 152 | ZNF621 |  |  | 1184 | 0.187 | 0.4130 | Yes |
| 153 | SLC2A1 |  |  | 1190 | 0.186 | 0.4141 | Yes |
| 154 | ZFYVE16 |  |  | 1206 | 0.184 | 0.4140 | Yes |
| 155 | CERS6 |  |  | 1208 | 0.183 | 0.4155 | Yes |
| 156 | HEPACAM |  |  | 1214 | 0.183 | 0.4166 | Yes |
| 157 | ZMAT1 |  |  | 1229 | 0.181 | 0.4166 | Yes |
| 158 | DNASE2 |  |  | 1252 | 0.179 | 0.4156 | Yes |
| 159 | PTAR1 |  |  | 1266 | 0.177 | 0.4157 | Yes |
| 160 | TFPI |  |  | 1278 | 0.175 | 0.4159 | Yes |
| 161 | SEMA6D |  |  | 1286 | 0.173 | 0.4167 | Yes |
| 162 | JAG1 |  |  | 1304 | 0.170 | 0.4162 | Yes |
| 163 | NLGN1 |  |  | 1305 | 0.170 | 0.4177 | Yes |
| 164 | OPHN1 |  |  | 1329 | 0.168 | 0.4166 | No |
| 165 | GLDC |  |  | 1341 | 0.167 | 0.4168 | No |
| 166 | MRPS5 |  |  | 1395 | 0.162 | 0.4120 | No |
| 167 | GJA1 |  |  | 1399 | 0.162 | 0.4131 | No |
| 168 | FOS |  |  | 1412 | 0.161 | 0.4131 | No |
| 169 | BMP7 |  |  | 1419 | 0.160 | 0.4139 | No |
| 170 | PSENEN |  |  | 1471 | 0.155 | 0.4093 | No |
| 171 | IFI27L1 |  |  | 1481 | 0.154 | 0.4096 | No |
| 172 | LPP |  |  | 1489 | 0.154 | 0.4102 | No |
| 173 | AXL |  |  | 1495 | 0.153 | 0.4109 | No |
| 174 | CLDND1 |  |  | 1501 | 0.152 | 0.4117 | No |
| 175 | LIFR |  |  | 1520 | 0.150 | 0.4110 | No |
| 176 | PTCH1 |  |  | 1526 | 0.150 | 0.4117 | No |
| 177 | TMEM170A |  |  | 1536 | 0.148 | 0.4120 | No |
| 178 | EGFR |  |  | 1547 | 0.147 | 0.4121 | No |
| 179 | CST3 |  |  | 1602 | 0.141 | 0.4071 | No |
| 180 | VEGFA |  |  | 1634 | 0.139 | 0.4047 | No |
| 181 | GABBR1 |  |  | 1652 | 0.138 | 0.4039 | No |
| 182 | SPARCL1 |  |  | 1663 | 0.136 | 0.4040 | No |
| 183 | PAQR8 |  |  | 1666 | 0.136 | 0.4049 | No |
| 184 | CSPG5 |  |  | 1670 | 0.136 | 0.4058 | No |
| 185 | SLC35A5 |  |  | 1680 | 0.135 | 0.4060 | No |
| 186 | DCLK2 |  |  | 1694 | 0.134 | 0.4056 | No |
| 187 | APC |  |  | 1704 | 0.133 | 0.4058 | No |
| 188 | MEIS1 |  |  | 1728 | 0.131 | 0.4043 | No |
| 189 | NTM |  |  | 1729 | 0.131 | 0.4054 | No |
| 190 | MLLT6 |  |  | 1733 | 0.131 | 0.4063 | No |
| 191 | HS2ST1 |  |  | 1744 | 0.130 | 0.4063 | No |
| 192 | NPAS3 |  |  | 1752 | 0.130 | 0.4066 | No |
| 193 | NCKAP5 |  |  | 1790 | 0.127 | 0.4034 | No |
| 194 | VPS36 |  |  | 1842 | 0.123 | 0.3985 | No |
| 195 | TMEM9B |  |  | 1845 | 0.123 | 0.3994 | No |
| 196 | CYR61 |  |  | 1850 | 0.123 | 0.4000 | No |
| 197 | HNRNPA2B1 |  |  | 1852 | 0.122 | 0.4010 | No |
| 198 | LRRC17 |  |  | 1859 | 0.122 | 0.4014 | No |
| 199 | RFTN2 |  |  | 1867 | 0.121 | 0.4016 | No |
| 200 | CSGALNACT1 |  |  | 1872 | 0.121 | 0.4023 | No |
| 201 | PCDH17 |  |  | 1889 | 0.120 | 0.4015 | No |
| 202 | HMGCS1 |  |  | 1899 | 0.118 | 0.4015 | No |
| 203 | CCDC80 |  |  | 1921 | 0.117 | 0.4000 | No |
| 204 | TNFRSF19 |  |  | 1943 | 0.116 | 0.3986 | No |
| 205 | CD99 |  |  | 1945 | 0.116 | 0.3995 | No |
| 206 | NLGN3 |  |  | 1947 | 0.115 | 0.4005 | No |
| 207 | NOTCH3 |  |  | 1978 | 0.113 | 0.3979 | No |
| 208 | WSCD1 |  |  | 1980 | 0.113 | 0.3988 | No |
| 209 | BMPR1A |  |  | 2003 | 0.111 | 0.3973 | No |
| 210 | MCCC2 |  |  | 2021 | 0.110 | 0.3963 | No |
| 211 | MEST |  |  | 2059 | 0.108 | 0.3929 | No |
| 212 | TGFB2 |  |  | 2112 | 0.104 | 0.3877 | No |
| 213 | RNPC3 |  |  | 2141 | 0.103 | 0.3853 | No |
| 214 | HES1 |  |  | 2302 | 0.092 | 0.3674 | No |
| 215 | PARP14 |  |  | 2313 | 0.091 | 0.3670 | No |
| 216 | IFT88 |  |  | 2320 | 0.091 | 0.3671 | No |
| 217 | LRP4 |  |  | 2410 | 0.084 | 0.3574 | No |
| 218 | KANK1 |  |  | 2429 | 0.083 | 0.3561 | No |
| 219 | PHGDH |  |  | 2485 | 0.081 | 0.3503 | No |
| 220 | IVD |  |  | 2515 | 0.078 | 0.3476 | No |
| 221 | TEAD1 |  |  | 2525 | 0.077 | 0.3472 | No |
| 222 | PBXIP1 |  |  | 2536 | 0.077 | 0.3468 | No |
| 223 | SLC7A11 |  |  | 2577 | 0.075 | 0.3427 | No |
| 224 | MYO6 |  |  | 2580 | 0.075 | 0.3432 | No |
| 225 | PARP9 |  |  | 2624 | 0.073 | 0.3388 | No |
| 226 | ABCA3 |  |  | 2662 | 0.070 | 0.3351 | No |
| 227 | BBS2 |  |  | 2698 | 0.068 | 0.3316 | No |
| 228 | ACOX1 |  |  | 2700 | 0.068 | 0.3320 | No |
| 229 | DPF3 |  |  | 2714 | 0.067 | 0.3311 | No |
| 230 | ADCY2 |  |  | 2745 | 0.066 | 0.3282 | No |
| 231 | CASC4 |  |  | 2783 | 0.063 | 0.3244 | No |
| 232 | PTPRD |  |  | 2784 | 0.063 | 0.3250 | No |
| 233 | SCCPDH |  |  | 2799 | 0.063 | 0.3239 | No |
| 234 | ITPR2 |  |  | 2803 | 0.063 | 0.3241 | No |
| 235 | PHYHIPL |  |  | 2821 | 0.062 | 0.3227 | No |
| 236 | MCC |  |  | 2841 | 0.061 | 0.3210 | No |
| 237 | EPB41L2 |  |  | 2855 | 0.060 | 0.3200 | No |
| 238 | ETV1 |  |  | 2867 | 0.059 | 0.3192 | No |
| 239 | PLEKHG1 |  |  | 2938 | 0.056 | 0.3115 | No |
| 240 | COL11A1 |  |  | 2950 | 0.055 | 0.3107 | No |
| 241 | ETV5 |  |  | 2957 | 0.055 | 0.3105 | No |
| 242 | SEL1L3 |  |  | 2958 | 0.055 | 0.3110 | No |
| 243 | ABAT |  |  | 3020 | 0.051 | 0.3043 | No |
| 244 | ARHGEF26 |  |  | 3032 | 0.050 | 0.3034 | No |
| 245 | ARHGAP31 |  |  | 3063 | 0.049 | 0.3004 | No |
| 246 | SLC38A3 |  |  | 3125 | 0.046 | 0.2936 | No |
| 247 | C2orf72 |  |  | 3156 | 0.044 | 0.2905 | No |
| 248 | ZNF521 |  |  | 3159 | 0.043 | 0.2906 | No |
| 249 | DPM3 |  |  | 3164 | 0.043 | 0.2905 | No |
| 250 | ABLIM1 |  |  | 3183 | 0.042 | 0.2888 | No |
| 251 | SAMD8 |  |  | 3190 | 0.042 | 0.2885 | No |
| 252 | MAST4 |  |  | 3192 | 0.041 | 0.2887 | No |
| 253 | DTNA |  |  | 3219 | 0.040 | 0.2860 | No |
| 254 | IGFBP5 |  |  | 3230 | 0.040 | 0.2852 | No |
| 255 | APCDD1 |  |  | 3295 | 0.037 | 0.2780 | No |
| 256 | SMC5 |  |  | 3298 | 0.036 | 0.2781 | No |
| 257 | ARAP2 |  |  | 3299 | 0.036 | 0.2784 | No |
| 258 | FUT11 |  |  | 3329 | 0.035 | 0.2754 | No |
| 259 | SLC25A18 |  |  | 3352 | 0.034 | 0.2731 | No |
| 260 | TCF7L2 |  |  | 3356 | 0.033 | 0.2730 | No |
| 261 | CHKA |  |  | 3386 | 0.032 | 0.2699 | No |
| 262 | ERAP1 |  |  | 3396 | 0.032 | 0.2691 | No |
| 263 | PURB |  |  | 3408 | 0.031 | 0.2681 | No |
| 264 | CTNNB1 |  |  | 3417 | 0.031 | 0.2675 | No |
| 265 | C3orf70 |  |  | 3474 | 0.028 | 0.2611 | No |
| 266 | METTL8 |  |  | 3491 | 0.028 | 0.2595 | No |
| 267 | RGMA |  |  | 3522 | 0.026 | 0.2562 | No |
| 268 | MED13 |  |  | 3533 | 0.026 | 0.2553 | No |
| 269 | DOCK1 |  |  | 3542 | 0.025 | 0.2546 | No |
| 270 | COG2 |  |  | 3569 | 0.024 | 0.2517 | No |
| 271 | SNAP23 |  |  | 3621 | 0.023 | 0.2459 | No |
| 272 | KCNJ16 |  |  | 3631 | 0.022 | 0.2451 | No |
| 273 | SPRED2 |  |  | 3653 | 0.022 | 0.2428 | No |
| 274 | AGPAT3 |  |  | 3684 | 0.021 | 0.2395 | No |
| 275 | LRRN3 |  |  | 3720 | 0.019 | 0.2355 | No |
| 276 | KAT2A |  |  | 3748 | 0.018 | 0.2325 | No |
| 277 | NBPF14 |  |  | 3751 | 0.018 | 0.2324 | No |
| 278 | LPIN2 |  |  | 3814 | 0.015 | 0.2253 | No |
| 279 | GULP1 |  |  | 3837 | 0.014 | 0.2228 | No |
| 280 | GAS1 |  |  | 3841 | 0.014 | 0.2226 | No |
| 281 | EFCAB2 |  |  | 3935 | 0.010 | 0.2118 | No |
| 282 | RHOJ |  |  | 3980 | 0.008 | 0.2067 | No |
| 283 | BDH2 |  |  | 4092 | 0.004 | 0.1937 | No |
| 284 | EEPD1 |  |  | 4100 | 0.003 | 0.1929 | No |
| 285 | SPRED1 |  |  | 4106 | 0.003 | 0.1923 | No |
| 286 | SIRPA |  |  | 4174 | -0.000 | 0.1844 | No |
| 287 | ROBO3 |  |  | 4187 | -0.001 | 0.1830 | No |
| 288 | DPP8 |  |  | 4214 | -0.002 | 0.1800 | No |
| 289 | ALDH7A1 |  |  | 4221 | -0.002 | 0.1793 | No |
| 290 | MAP3K1 |  |  | 4259 | -0.004 | 0.1750 | No |
| 291 | USP46 |  |  | 4260 | -0.004 | 0.1750 | No |
| 292 | PARD3 |  |  | 4265 | -0.004 | 0.1746 | No |
| 293 | MTMR10 |  |  | 4266 | -0.004 | 0.1746 | No |
| 294 | SOX2 |  |  | 4271 | -0.005 | 0.1742 | No |
| 295 | SOX8 |  |  | 4306 | -0.006 | 0.1703 | No |
| 296 | RASSF4 |  |  | 4325 | -0.007 | 0.1682 | No |
| 297 | ZC3HAV1 |  |  | 4394 | -0.010 | 0.1603 | No |
| 298 | SLC48A1 |  |  | 4396 | -0.010 | 0.1603 | No |
| 299 | RHBDD2 |  |  | 4523 | -0.015 | 0.1456 | No |
| 300 | PIK3R1 |  |  | 4545 | -0.016 | 0.1433 | No |
| 301 | SSPN |  |  | 4582 | -0.017 | 0.1392 | No |
| 302 | SEPT8 |  |  | 4589 | -0.018 | 0.1387 | No |
| 303 | CAMK2G |  |  | 4597 | -0.018 | 0.1380 | No |
| 304 | TOB2 |  |  | 4710 | -0.022 | 0.1250 | No |
| 305 | IQSEC1 |  |  | 4764 | -0.024 | 0.1190 | No |
| 306 | FAM181B |  |  | 4789 | -0.025 | 0.1164 | No |
| 307 | ID4 |  |  | 4830 | -0.027 | 0.1120 | No |
| 308 | HEATR5A |  |  | 4837 | -0.027 | 0.1115 | No |
| 309 | CTNND2 |  |  | 4868 | -0.028 | 0.1083 | No |
| 310 | APOL2 |  |  | 5005 | -0.033 | 0.0926 | No |
| 311 | UGP2 |  |  | 5013 | -0.034 | 0.0921 | No |
| 312 | TRIM9 |  |  | 5014 | -0.034 | 0.0924 | No |
| 313 | ABCD3 |  |  | 5045 | -0.034 | 0.0891 | No |
| 314 | PTPN13 |  |  | 5065 | -0.035 | 0.0872 | No |
| 315 | MYO10 |  |  | 5109 | -0.037 | 0.0825 | No |
| 316 | CA2 |  |  | 5139 | -0.038 | 0.0794 | No |
| 317 | TAOK3 |  |  | 5204 | -0.041 | 0.0723 | No |
| 318 | SRGAP2B |  |  | 5207 | -0.041 | 0.0724 | No |
| 319 | THG1L |  |  | 5227 | -0.041 | 0.0706 | No |
| 320 | ABI1 |  |  | 5375 | -0.048 | 0.0537 | No |
| 321 | ATP1B2 |  |  | 5376 | -0.048 | 0.0541 | No |
| 322 | KAT2B |  |  | 5388 | -0.048 | 0.0533 | No |
| 323 | MAPK1 |  |  | 5469 | -0.051 | 0.0443 | No |
| 324 | OGFRL1 |  |  | 5490 | -0.052 | 0.0425 | No |
| 325 | PHLPP1 |  |  | 5506 | -0.053 | 0.0412 | No |
| 326 | PCCB |  |  | 5554 | -0.055 | 0.0362 | No |
| 327 | TACC1 |  |  | 5576 | -0.056 | 0.0342 | No |
| 328 | PHACTR2 |  |  | 5613 | -0.058 | 0.0305 | No |
| 329 | FMNL2 |  |  | 5646 | -0.060 | 0.0273 | No |
| 330 | FERMT2 |  |  | 5664 | -0.061 | 0.0258 | No |
| 331 | ALDH6A1 |  |  | 5682 | -0.061 | 0.0244 | No |
| 332 | CAMK2D |  |  | 5690 | -0.062 | 0.0241 | No |
| 333 | SUCLG2 |  |  | 5712 | -0.062 | 0.0222 | No |
| 334 | QKI |  |  | 5715 | -0.062 | 0.0225 | No |
| 335 | ARHGAP5 |  |  | 5716 | -0.063 | 0.0231 | No |
| 336 | SWAP70 |  |  | 5773 | -0.064 | 0.0171 | No |
| 337 | EFCAB14 |  |  | 5776 | -0.065 | 0.0174 | No |
| 338 | NACC2 |  |  | 5780 | -0.065 | 0.0177 | No |
| 339 | SNTA1 |  |  | 5793 | -0.065 | 0.0168 | No |
| 340 | FAM19A5 |  |  | 5803 | -0.066 | 0.0164 | No |
| 341 | AHCYL1 |  |  | 5817 | -0.066 | 0.0154 | No |
| 342 | SASH1 |  |  | 5850 | -0.067 | 0.0123 | No |
| 343 | ARHGEF6 |  |  | 5891 | -0.069 | 0.0082 | No |
| 344 | MTSS1L |  |  | 5908 | -0.070 | 0.0069 | No |
| 345 | PDLIM3 |  |  | 5938 | -0.071 | 0.0042 | No |
| 346 | PRTFDC1 |  |  | 5982 | -0.073 | -0.0002 | No |
| 347 | ADD3 |  |  | 5984 | -0.073 | 0.0003 | No |
| 348 | IRF2 |  |  | 6031 | -0.076 | -0.0044 | No |
| 349 | GATM |  |  | 6035 | -0.076 | -0.0041 | No |
| 350 | ZNF639 |  |  | 6107 | -0.080 | -0.0117 | No |
| 351 | RHOBTB3 |  |  | 6115 | -0.081 | -0.0118 | No |
| 352 | CRYL1 |  |  | 6120 | -0.081 | -0.0115 | No |
| 353 | PDLIM5 |  |  | 6127 | -0.081 | -0.0115 | No |
| 354 | WDR41 |  |  | 6237 | -0.087 | -0.0235 | No |
| 355 | LARS |  |  | 6288 | -0.090 | -0.0286 | No |
| 356 | HIPK2 |  |  | 6320 | -0.091 | -0.0314 | No |
| 357 | LIMA1 |  |  | 6339 | -0.092 | -0.0327 | No |
| 358 | ITPK1 |  |  | 6350 | -0.093 | -0.0330 | No |
| 359 | PPP2R5A |  |  | 6380 | -0.094 | -0.0356 | No |
| 360 | TLK1 |  |  | 6386 | -0.094 | -0.0354 | No |
| 361 | SNX5 |  |  | 6418 | -0.097 | -0.0381 | No |
| 362 | NDRG4 |  |  | 6429 | -0.097 | -0.0384 | No |
| 363 | CNDP2 |  |  | 6466 | -0.100 | -0.0418 | No |
| 364 | TJP1 |  |  | 6500 | -0.101 | -0.0447 | No |
| 365 | HSDL2 |  |  | 6505 | -0.101 | -0.0443 | No |
| 366 | TRIM47 |  |  | 6510 | -0.102 | -0.0438 | No |
| 367 | PEX2 |  |  | 6580 | -0.107 | -0.0510 | No |
| 368 | GNG7 |  |  | 6584 | -0.107 | -0.0504 | No |
| 369 | OLIG2 |  |  | 6595 | -0.108 | -0.0506 | No |
| 370 | ZIC1 |  |  | 6618 | -0.109 | -0.0522 | No |
| 371 | OAT |  |  | 6648 | -0.111 | -0.0546 | No |
| 372 | NEK1 |  |  | 6679 | -0.112 | -0.0571 | No |
| 373 | NADK2 |  |  | 6686 | -0.113 | -0.0568 | No |
| 374 | WWC2 |  |  | 6751 | -0.117 | -0.0633 | No |
| 375 | GSTM4 |  |  | 6789 | -0.120 | -0.0665 | No |
| 376 | CHP1 |  |  | 6793 | -0.120 | -0.0658 | No |
| 377 | FYN |  |  | 6823 | -0.123 | -0.0681 | No |
| 378 | BAALC |  |  | 6848 | -0.125 | -0.0698 | No |
| 379 | TGIF1 |  |  | 6926 | -0.131 | -0.0777 | No |
| 380 | SESN3 |  |  | 6935 | -0.132 | -0.0774 | No |
| 381 | ZEB1 |  |  | 6944 | -0.132 | -0.0772 | No |
| 382 | IMPACT |  |  | 6953 | -0.133 | -0.0769 | No |
| 383 | ZMAT3 |  |  | 6960 | -0.134 | -0.0764 | No |
| 384 | CHPT1 |  |  | 6977 | -0.135 | -0.0771 | No |
| 385 | CDCA7L |  |  | 7034 | -0.140 | -0.0824 | No |
| 386 | TXNIP |  |  | 7037 | -0.140 | -0.0814 | No |
| 387 | CREM |  |  | 7054 | -0.142 | -0.0820 | No |
| 388 | ATF7 |  |  | 7056 | -0.142 | -0.0808 | No |
| 389 | ZCCHC24 |  |  | 7078 | -0.143 | -0.0820 | No |
| 390 | GFAP |  |  | 7113 | -0.146 | -0.0847 | No |
| 391 | EFHD2 |  |  | 7122 | -0.147 | -0.0843 | No |
| 392 | HADHB |  |  | 7132 | -0.148 | -0.0840 | No |
| 393 | DDAH1 |  |  | 7257 | -0.162 | -0.0972 | No |
| 394 | DUSP6 |  |  | 7258 | -0.162 | -0.0957 | No |
| 395 | ARHGAP12 |  |  | 7261 | -0.163 | -0.0945 | No |
| 396 | SEPT2 |  |  | 7297 | -0.167 | -0.0971 | No |
| 397 | ZFP36L2 |  |  | 7317 | -0.169 | -0.0978 | No |
| 398 | ID2 |  |  | 7333 | -0.171 | -0.0980 | No |
| 399 | IDI1 |  |  | 7337 | -0.172 | -0.0968 | No |
| 400 | HEY1 |  |  | 7370 | -0.177 | -0.0990 | No |
| 401 | RGS20 |  |  | 7387 | -0.180 | -0.0993 | No |
| 402 | LHX2 |  |  | 7455 | -0.187 | -0.1055 | No |
| 403 | SPRY2 |  |  | 7461 | -0.187 | -0.1044 | No |
| 404 | ZFP36L1 |  |  | 7466 | -0.188 | -0.1031 | No |
| 405 | FAM120A |  |  | 7521 | -0.193 | -0.1078 | No |
| 406 | UBL3 |  |  | 7525 | -0.194 | -0.1064 | No |
| 407 | GLUD1 |  |  | 7545 | -0.197 | -0.1068 | No |
| 408 | PSMD10 |  |  | 7550 | -0.197 | -0.1055 | No |
| 409 | GLUL |  |  | 7594 | -0.203 | -0.1087 | No |
| 410 | OLFM2 |  |  | 7624 | -0.207 | -0.1103 | No |
| 411 | TRAPPC3 |  |  | 7692 | -0.217 | -0.1162 | No |
| 412 | PSAT1 |  |  | 7709 | -0.220 | -0.1161 | No |
| 413 | TRIB2 |  |  | 7741 | -0.224 | -0.1177 | No |
| 414 | AIF1L |  |  | 7762 | -0.227 | -0.1181 | No |
| 415 | SRPK2 |  |  | 7766 | -0.227 | -0.1164 | No |
| 416 | ID1 |  |  | 7821 | -0.238 | -0.1206 | No |
| 417 | CLNS1A |  |  | 7824 | -0.239 | -0.1187 | No |
| 418 | PSME2 |  |  | 7885 | -0.252 | -0.1234 | No |
| 419 | RASSF2 |  |  | 7903 | -0.256 | -0.1231 | No |
| 420 | NDUFA4 |  |  | 7910 | -0.257 | -0.1215 | No |
| 421 | JUN |  |  | 7930 | -0.262 | -0.1214 | No |
| 422 | MT1X |  |  | 7950 | -0.266 | -0.1212 | No |
| 423 | PDXK |  |  | 7966 | -0.269 | -0.1206 | No |
| 424 | BBX |  |  | 7987 | -0.272 | -0.1205 | No |
| 425 | SEPT7 |  |  | 8018 | -0.279 | -0.1215 | No |
| 426 | PMP2 |  |  | 8058 | -0.288 | -0.1235 | No |
| 427 | LAP3 |  |  | 8062 | -0.288 | -0.1213 | No |
| 428 | NDUFB5 |  |  | 8071 | -0.291 | -0.1196 | No |
| 429 | SAT1 |  |  | 8162 | -0.314 | -0.1273 | No |
| 430 | MPC1 |  |  | 8166 | -0.316 | -0.1249 | No |
| 431 | NEK6 |  |  | 8190 | -0.323 | -0.1247 | No |
| 432 | FHL1 |  |  | 8198 | -0.326 | -0.1226 | No |
| 433 | ADI1 |  |  | 8226 | -0.332 | -0.1227 | No |
| 434 | HOPX |  |  | 8257 | -0.341 | -0.1232 | No |
| 435 | STK17A |  |  | 8267 | -0.345 | -0.1212 | No |
| 436 | MT3 |  |  | 8279 | -0.348 | -0.1193 | No |
| 437 | LITAF |  |  | 8324 | -0.361 | -0.1213 | No |
| 438 | SOX9 |  |  | 8327 | -0.361 | -0.1182 | No |
| 439 | GNG12 |  |  | 8328 | -0.362 | -0.1150 | No |
| 440 | VEGFB |  |  | 8352 | -0.370 | -0.1144 | No |
| 441 | LDHB |  |  | 8372 | -0.379 | -0.1132 | No |
| 442 | ENHO |  |  | 8432 | -0.400 | -0.1165 | No |
| 443 | NDUFB3 |  |  | 8447 | -0.406 | -0.1145 | No |
| 444 | LYPD1 |  |  | 8476 | -0.422 | -0.1140 | No |
| 445 | GCSH |  |  | 8484 | -0.426 | -0.1110 | No |
| 446 | PEA15 |  |  | 8509 | -0.439 | -0.1099 | No |
| 447 | SCP2 |  |  | 8513 | -0.442 | -0.1063 | No |
| 448 | CXXC5 |  |  | 8526 | -0.446 | -0.1037 | No |
| 449 | PRDX1 |  |  | 8584 | -0.476 | -0.1061 | No |
| 450 | RAB13 |  |  | 8590 | -0.481 | -0.1024 | No |
| 451 | ANXA5 |  |  | 8626 | -0.510 | -0.1019 | No |
| 452 | MRFAP1 |  |  | 8652 | -0.527 | -0.1001 | No |
| 453 | VIM |  |  | 8663 | -0.535 | -0.0964 | No |
| 454 | SLC25A5 |  |  | 8681 | -0.546 | -0.0935 | No |
| 455 | SRI |  |  | 8709 | -0.572 | -0.0916 | No |
| 456 | POLR2L |  |  | 8715 | -0.578 | -0.0870 | No |
| 457 | ENO1 |  |  | 8734 | -0.593 | -0.0837 | No |
| 458 | S100A16 |  |  | 8739 | -0.596 | -0.0789 | No |
| 459 | MT2A |  |  | 8740 | -0.598 | -0.0735 | No |
| 460 | CNN3 |  |  | 8752 | -0.613 | -0.0693 | No |
| 461 | SPATS2L |  |  | 8793 | -0.645 | -0.0682 | No |
| 462 | FABP7 |  |  | 8811 | -0.661 | -0.0642 | No |
| 463 | S100A13 |  |  | 8820 | -0.670 | -0.0591 | No |
| 464 | RHOC |  |  | 8823 | -0.676 | -0.0533 | No |
| 465 | FABP5 |  |  | 8829 | -0.684 | -0.0477 | No |
| 466 | RAB31 |  |  | 8831 | -0.687 | -0.0417 | No |
| 467 | TOMM7 |  |  | 8861 | -0.724 | -0.0386 | No |
| 468 | GNG5 |  |  | 8866 | -0.728 | -0.0325 | No |
| 469 | RHOA |  |  | 8869 | -0.734 | -0.0261 | No |
| 470 | SNX3 |  |  | 8881 | -0.748 | -0.0207 | No |
| 471 | DBI |  |  | 8895 | -0.764 | -0.0154 | No |
| 472 | S100B |  |  | 8908 | -0.784 | -0.0097 | No |
| 473 | PKM |  |  | 8959 | -0.915 | -0.0074 | No |
| 474 | LGALS1 |  |  | 8982 | -1.135 | 0.0002 | No |
Table: GSEA details [plain text format]

  

Fig 2: RICHARDS\_NOWAKOWSKI\_SCIENCE\_2017\_ASTROCYTE\_UPREG: Random ES distribution      
 Gene set null distribution of ES for **RICHARDS\_NOWAKOWSKI\_SCIENCE\_2017\_ASTROCYTE\_UPREG**
